# Hyperpolarising Pyruvate through Signal Amplification by Reversible Exchange (SABRE)

**DOI:** 10.1002/anie.201905483

**Published:** 2019-06-17

**Authors:** Wissam Iali, Soumya S. Roy, Ben J. Tickner, Fadi Ahwal, Aneurin J. Kennerley, Simon B. Duckett

**Affiliations:** ^1^ Centre for Hyperpolarisation in Magnetic Resonance (CHyM) Department of Chemistry University of York Heslington York YO10 5NY UK; ^2^ Present address: Department of Inorganic and Physical Chemistry Indian Institute of Science Bangalore 560012 India

**Keywords:** catalysis, hyperpolarization, pyruvate, SABRE, singlet state

## Abstract

Hyperpolarisation methods that premagnetise agents such as pyruvate are currently receiving significant attention because they produce sensitivity gains that allow disease tracking and interrogation of cellular metabolism by magnetic resonance. Here, we communicate how signal amplification by reversible exchange (SABRE) can provide strong ^13^C pyruvate signal enhancements in seconds through the formation of the novel polarisation transfer catalyst [Ir(H)_2_(η^2^‐pyruvate)(DMSO)(IMes)]. By harnessing SABRE, strong signals for [1‐^13^C]‐ and [2‐^13^C]pyruvate in addition to a long‐lived singlet state in the [1,2‐^13^C_2_] form are readily created; the latter can be observed five minutes after the initial hyperpolarisation step. We also demonstrate how this development may help with future studies of chemical reactivity.

## Graphical abstract

**Signal amplification** by reversible exchange (SABRE) can provide strong ^13^C pyruvate signal enhancements in seconds through the formation of the novel polarisation transfer catalyst [Ir(H)_2_(η^2^‐pyruvate)(DMSO)(IMes)]. By harnessing SABRE, strong signals for [1‐^13^C]‐ and [2‐^13^C]pyruvate are readily created.

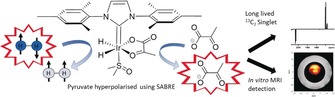

Pyruvate lies at the junction of many metabolic processes in living cells, being produced from glucose before it enters cellular energy production pathways.^1^ Pyruvate is converted into lactate under the anaerobic conditions associated with cancer, offering a route to diagnose cellular abnormalities.^2^ Clinical trials are progressing that harness this approach for the diagnosis of cancer by magnetic resonance imaging (MRI).^3^ These developments build from decades of research into hyperpolarisation techniques such as dynamic nuclear polarisation (DNP),^4^ the method that has allowed the hyperpolarisation and subsequent in vivo detection of such biomolecules by MRI.3b, 3c, 5

Hyperpolarisation can also be created by parahydrogen (*p*‐H_2_) induced polarisation (PHIP). This approach utilises the reactivity and nuclear spin orientations of *p*‐H_2_ to create highly visible hydrogenation products.^6^ Molecules hyperpolarised using PHIP have been widely used in NMR spectroscopy, and there are examples that feature in vivo imaging.^5^, 6c, 7 While pyruvate has no readily accessible unsaturated precursor suitable for hydrogenation using PHIP, Aime and co‐workers have developed an elegant route to form aqueous solutions of hyperpolarised pyruvate by incorporation of a rapidly hydrogenated and subsequently hydrolysed side arm.^8^

Here though, we hyperpolarise pyruvate based on the non‐hydrogenative PHIP derived signal amplification by reversible exchange (SABRE) process, which is shown in Figure 1.^9^ The chemical identity of pyruvate is unaffected by this process, which has already achieved substantial levels of polarisation (63 % in ^1^H,^10^ 25 % in ^13^C,^11^ and 43 % in ^15^N^12^) in a range of materials that predominantly bind to a metal polarisation transfer catalyst through nitrogen centres.^13^

**Figure 1:**
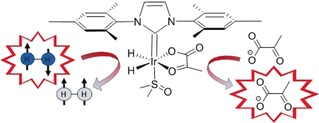
Schematic depiction of the SABRE hyperpolarisation process in which parahydrogen is used to hyperpolarise pyruvate via the polarisation transfer catalyst [Ir(H)_2_(η^2^‐pyruvate)(DMSO)(IMes)].

SABRE harnesses *p*‐H_2_, which is one of a growing range of molecules that exists as a nuclear spin singlet. When this molecule binds to a transition‐metal centre to form a dihydride complex, it is possible to transfer polarisation from *p*‐H_2_ into a ligand if there are different hydrides coupling to the nucleus that receive it. The potentially long lifetime of such states makes them ideal fuels for polarisation transfer^14^ or indeed later detection.13a, 13e, 15 *p*‐H_2_ can be formed in >95 % purity by simply cooling H_2_ gas to <40 K in the presence of a suitable conversion catalyst.^16^

In a remarkable development, Levitt and co‐workers created a molecular spin singlet state by radiofrequency excitation in 2004,^17^ and more recently demonstrated the existence of a coupled spin‐1/2 nuclear ^13^C pair with a singlet‐state lifetime that exceeds one hour.^18^ Levitt created a 1,2‐^13^C_2_ singlet state in pyruvate using DNP15b, 19 that had a reported lifetime of 70 s. Here, we use SABRE to create pyruvate hyperpolarisation, including the singlet form of the 1,2‐^13^C_2_ isotopologue in a process that proceeds spontaneously without the need for complex instrumentation or pulse sequences.

In order to achieve this goal, we first had to overcome the challenge of weak iridium pyruvate binding, which prevents typical hyperpolarisation of pyruvate using SABRE. The presence of an appropriate dimethyl sulfoxide (DMSO) co‐ligand allows for the assembly of a highly reactive polarisation transfer catalyst that overcomes poor pyruvate ligation. This is achieved by the reaction of [Ir(Cl)(COD)(IMes)] (IMes=1,3‐bis(2,4,6‐trimethylphenyl)imidazol‐2‐ylidene, COD=*cis*,*cis*‐1,5‐cyclooctadiene), the well‐known SABRE catalyst precursor,^20^ with DMSO, H_2_, and sodium pyruvate to form [Ir(H)_2_(η^2^‐pyruvate)(DMSO)(IMes)]. The resulting high‐field NMR spectra and characterisation data that confirm this product formation are detailed in the Supporting Information, and its structure is represented in Figure 1. The [1‐^13^C]‐ (**1**), [2‐^13^C]‐ (**2**), and [1,2‐^13^C_2_]‐pyruvate (**3**) isotopologues of sodium pyruvate (**4**) are used in this work.

Because of the low symmetry of pyruvate, [Ir(H)_2_(η^2^‐pyruvate)(DMSO)(IMes)] yields two inequivalent hydride ligands such that at low magnetic fields, an [AA′B] spin system is formed. Modelling the propagation of hyperpolarisation from the *p*‐H_2_‐derived hydride ligands of this product into the bound pyruvate ^13^C nuclei predicts optimum polarisation transfer at a magnetic field strength of ±(−*J*_HH_+*J*_HC*_)/Δ*γ* 13a, 21 (see the Supporting Information). Here, *J*_HH_ corresponds to the *J* coupling between the hydride ligands, *J*_HC*_ is (*J*_HC_+*J*_H′C_)/2, the combined hydride–carbon cross‐coupling in the complex, and Δ*γ* is the difference in magnetogyric ratios (*γ*_H_−*γ*_C_) of proton and carbon nuclei. The predicted field strengths are about 100 times lower than the Earth's natural magnetic field and were achieved experimentally by housing the sample in a mu‐metal shield in conjunction with a field top‐up solenoid as detailed in the Supporting Information.^22^ This approach reflects a variant of SABRE that has been called SABRE‐SHEATH.^22^, ^23^

A sample of **1**, [IrCl(COD)(IMes)] precatalyst, and DMSO with 3 bar *p*‐H_2_ was therefore prepared. This was shaken in the mu‐metal‐shielded solenoid for 20 s at 9 mG. Upon transfer into a 9.4 T magnet, strongly hyperpolarised ^13^C resonances corresponding to free pyruvate were observed. Subsequently, polarisation transfer between +20 to −21 mG confirmed that ±9 mG reflects the maximum signal intensity as portrayed by both Figure 2 a and the model. An overall ^13^C polarisation level of about 1 % results for a sample containing [Ir(Cl)(COD)(IMes)] (5 mm), DMSO, and the corresponding pyruvate isotopologue in a 1:8:5 ratio after shaking with 3 bar of *p*‐H_2_ for 20 s. When a similar sample containing **2** was examined, the corresponding maximum polarisation level proved to be 0.6 % when transfer took place at ±3 mG. This reduction in efficiency is due to a smaller *J* ${}_{{}^{1}H-{}^{13}C}$ transfer coupling and shorter spin‐state lifetime (see Table 1), which results in more efficient signal decay during the slow polarisation transfer step.^24^ Close examination of the hyperpolarised ^13^C NMR spectra of **1** and **2** revealed peaks for catalyst‐bound pyruvate in [Ir(H)_2_(η^2^‐pyruvate)(DMSO)(IMes)] and [1,2‐^13^C_2_]pyruvate (**3**) as illustrated in Figure 2 b. The detection of **3**, present at 1.1 % natural abundance, confirms the impressive nature of the signal amplification that is achieved. The ^13^C relaxation times and polarisation levels of these species are detailed in Table 1.15b, 19 Values of 32.5±4.7 s and 18.2±3.0 s were obtained, respectively, for the in high‐field relaxation times of the ^13^C signals of **1** and **2**, which are close to the normal values of 35.4±0.5 s and 20.1±0.5 s, respectively. Hence, the presence of the SABRE catalyst in these solutions does not significantly change their value. This reflects the fact that magnetisation build up by this catalyst is slow due to slow ligand exchange and small propagating *J*_HC_ values, which differs from the typical response achieved when nitrogen‐containing heterocycles are examined.^10^, ^25^

**Figure 2:**
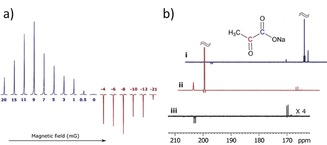
SABRE‐hyperpolarised NMR spectra of pyruvate. A) Plot showing how the intensity of the ^13^C NMR response of **1** varies with the magnetic field that the sample experiences during polarisation transfer; maximum polarisation transfer efficiency (signal intensity) is achieved with a ca. 9 mG field. B) SABRE‐hyperpolarised ^13^C NMR spectra of i) **1** and ii) **2** after transfer in a 5 mG mixing field, which show strongly enhanced signals from the labelled carbon atoms. These spectra also reveal a singlet response originating from the 1.1 % of **3** present; iii) corresponding spectrum of **1** after hyperpolarisation transfer in the Earth's field (ca. 500 mG), which selects signals from **3**.

**Table 1:** Hyperpolarisation levels and lifetimes for isotopologues **1**–**4**.

Substrate	Net ^13^C polarisation [%]	Thermally polarised lifetimes *T*_1_ [s]	Hyperpolarised lifetimes *T*_1_ and *T*_LLS_ [s]
**1**	C_1_: 0.96	C_1_: 35.4±0.5	C_1_ *T*_1_: 32.5±4.7
**2**	C_2_: 0.60	C_2_: 20.1±0.5	C_2_ *T*_1_: 18.2±3.0
**3**	C_1_: 1.85 C_2_: 1.65	C_1_: 33.6±0.5 C_2_: 21.2±0.4	*T*_LLS_ (HF): 43.5±0.8 *T*_LLS_ (LF): 85.4±8.5
**4**	C_1_: 0.55 C_2_: 0.35 C_3_: 0.22	C_1_: 31.4±1.2 C_2_: 8.6±1.5 C_3_: 3.3±0.7	C_1_ *T*_1_: 28.5±5.5 C_2_ *T*_1_: 15.7±1.9 C_3_ *T*_1_: 3.0±2.5

In contrast to the situation with **1** and **2**, isotopomer **3** is predicted to give a singlet‐based response after polarisation transfer between 0 G and 1 kG (Figure 3 a, b).13a This reflects the fact that the metal dihydride complex that results is now of the [AA′BB′] type at low field.^22^ This prediction was confirmed experimentally by monitoring the effect of excitation angle on the resulting signal profile as shown in Figure 3 c, d. A close fit between the experimental and predicted data is observed. Furthermore, the singlet state forms with an amplitude that is 1740 times larger than the normal Zeeman polarisation observed in this sample when detected at 11.75 T, which reflects a purity of 1.75 % relative to it; the sample contained [Ir(Cl)(COD)(IMes)] (5 mm), DMSO, and **3** in a ratio of 1:8:5. The lifetime of this magnetisation was assessed after sample storage at both low and high field as a function of catalyst loading (see typical plots in Figure 4). These high‐field observations (Figure 4 a) indicate that cross‐relaxation‐induced polarisation transfer occurs within the spin system during this period that makes the originally weaker resonance components increase in intensity (Figure 4 b). The corresponding magnetic state lifetime is 85.4±8.5 s with low‐field storage (0.5 G) but at 11.7 T it is reduced to 43.5±0.8 s as a consequence of the change in characteristics of the underlying spin states. Consequently, harnessing states that start out as a singlet, rather than the quicker relaxing Zeeman terms, may improve the duration of signal visibility. This may provide interesting applications for SABRE hyperpolarised pyruvate. While the polarisation levels that we demonstrate here are not as high as those that have been reported using PHIP‐SAH^8^ and DNP,^1^, 3b, 3c, 19, 26 this route can create singlet hyperpolarisation in a refreshable, lower‐cost alternative technique that may provide significant advantages in the future.

**Figure 3:**
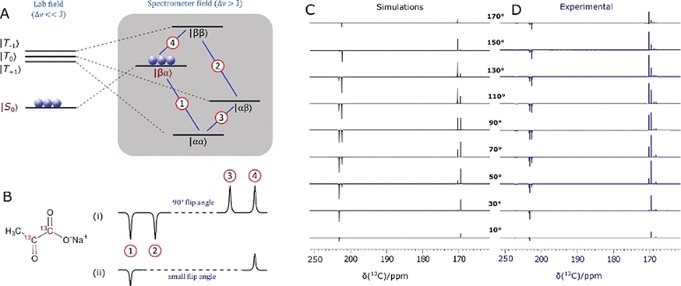
Demonstrating singlet character. A) Energy level diagram for the evolution of the two spin‐1/2 1,2‐^13^C_2_ coupled spin system of **3** after hyperpolarisation and sample movement from low (left) to high field (right). B) Simulated NMR spectra resulting from i) 90° and ii) low flip angle excitations. C) Simulated and D) experimentally observed ^13^C NMR signal patterns for **3** after transfer in the Earth's field (ca. 500 mG) as a function of flip angle pulse duration (10° to 170° in steps of 20°) to confirm singlet state formation.

**Figure 4:**
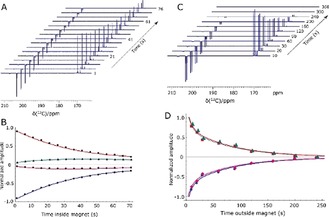
Determination of singlet lifetime. A) Series of hyperpolarised ^13^C NMR spectra of **3** acquired 1 s to 76 s after sample insertion into the spectrometer with a 9° flip angle pulse. B) The corresponding signal intensity plot whose fitting (solid lines) yields a high‐field (HF) lifetime, *T*_LLS_, of 43.5±0.8 s. Similar data is shown in (C) and (D) after sample storage at 0.5 G (LF) time. These points yield a *T*_LLS_ (LF) value of 85.4±8.5 s.

We demonstrate in Figure 5 that in vitro MRI detection of a 0.4 mm sample of **3** in a 70:30 D_2_O/[D_6_]ethanol mixture is possible. We also show a future potential use in hyperpolarised reaction monitoring in Figure 6. Here, hydrogen peroxide is added to a solution of SABRE‐hyperpolarised **3** in 70:30 D_2_O/[D_6_]ethanol, and signals for hyperpolarised ethanoic acid and carbon dioxide are seen, which encode concentration changes that take place over 25 s.

**Figure 5:**
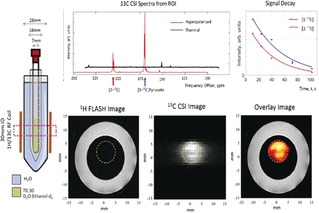
Hyperpolarised image detection. Hyperpolarised ^13^C FLASH image (orange) overlaid onto a ^1^H FLASH image of water (white outer ring) originating from the sample (left). The ^13^C CSI image reflects a 32×32 mm region of this hyperpolarised solution at 1 mm resolution. The data was collected at 9.4 T using single‐shot FLASH and EPI measurements and confirms that MRI detection is possible with these enhancement levels.

**Figure 6:**
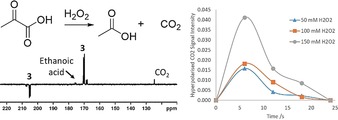
Hyperpolarised reaction monitoring. Resonances corresponding to ethanoic acid and CO_2_ appear after the addition of H_2_O_2_ to solutions of SABRE‐hyperpolarised pyruvate. This reaction was monitored by recording a series of hyperpolarised ^13^C NMR spectra.

In conclusion, we have presented an approach to hyperpolarise pyruvate directly by SABRE in just a few seconds. This simple process harnesses a solenoid housed within a mu‐metal shield to optimise the polarisation transfer pathway within a novel [Ir(H)_2_(η^2^‐pyruvate)(IMes)(DMSO)] catalyst. The hyperpolarised ^13^C signals appear with intensities that are three orders of magnitude above their thermal values. Furthermore, when [1,2‐^13^C_2_]pyruvate is employed, the ready formation of a long‐lived singlet state with a low field lifetime of 85.4±8.5 s is achieved. We demonstrate that these polarisation levels allow in vitro images to be collected of a 70:30 D_2_O/[D_6_]ethanol mixture and a simple organic transformation to be viewed. We expect that the signal gain can be improved by optimisation of the ligand exchange processes within the polarisation transfer catalyst and by using higher pressures of *p*‐H_2_ with continuous bubbling.^22^, ^27^ Furthermore, it is clear that the ligand sphere of the metal catalyst controls substrate binding. We therefore expect further refinements of this “co‐ligand” approach to extend SABRE to a much wider range of substrates that bind through oxygen or nitrogen. Hence this work reflects an important step in the future development of SABRE.

## Conflict of interest

W.I., S.S.R., B.J.T., and S.B.D. are inventors on a University of York patent application on this work (Patent No. GB1818171.9, filed 7 November 2018).

## Supporting information

As a service to our authors and readers, this journal provides supporting information supplied by the authors. Such materials are peer reviewed and may be re‐organized for online delivery, but are not copy‐edited or typeset. Technical support issues arising from supporting information (other than missing files) should be addressed to the authors.

Supplementary
