# Supplementary material for: Hyperpolarising Pyruvate through Signal Amplification by Reversible Exchange (SABRE)
Source: Angew Chem Int Ed Engl. 2019 Jun 17;58(30):10271–5. doi: 10.1002/anie.201905483 (PMC7004201; doi:10.1002/anie.201905483)
Supplement: Supplementary file 1 — Supplementary [file ANIE-58-10271-s001.pdf]

Angewandte Chemie
Eine Zeitschrift der Gesellschaft Deutscher Chemiker

Supporting Information

Hyperpolarising Pyruvate through Signal Amplification by Reversible Exchange (SABRE)

*Wissam Iali[+], Soumya S. Roy[+], Ben J. Tickner, Fadi Ahwal, Aneurin J. Kennerley, and Simon B. Duckett**

anie_201905483_sm_miscellaneous_information.pdf

Author Contributions

F.A. designed the equipment.

Supplementary Material

S1. Experimental Procedures

S2. NMR characterization

S3. Theory and simulations

S4. Hyperpolarized NMR spectra examples

S5. Mu-metal shielded solenoid

S6. References

S1. Experimental procedures

Chemicals. Sodium-1-pyruvate-[^{13}C] (**1**), sodium-2-pyruvate-[^{13}C] (**2**), sodium-1,2-pyruvate-[$^{13}C_2$] (**3**), sodium pyruvate (**4**) and deuterated solvents were purchased from Sigma-Aldrich and used without any further purification. The [IrCl(COD)(IMes)] catalyst precursor was synthesized in our laboratory according to a literature procedure.[1]

Sample preparation. Samples were prepared with a fixed ratio of substrate to catalyst and DMSO in 0.6 mL of methanol-*d*$_4$ in a 5 mm NMR tube that was fitted with J. Young's tap. The solutions were degassed by the freeze-pump-thaw technique and subsequently filled with *p*-H_2 gas to a 3 bar pressure. A typical ratio of substrate, catalyst (5mM) and DMSO is 5:1:8. Para-hydrogen. Hydrogen gas was enriched to over 93% para-hydrogen in the presence of a spin-exchange catalyst (Fe_2O_3) at low temperature (36 K) and added to the NMR tubes used in these SABRE experiments.

SABRE Experiments. Two different routes were used to achieve ^{13}C SABRE hyperpolarization – (1) direct magnetization transfer at a specified magnetic field and (2) hyperpolarized singlet order creation in a non-zero field. In the former case, a solenoid coil is placed inside a mu-metal shield to achieve the desired matching field. This solenoid had length 18 cm and 100 turns. The single layered mu-metal cylinder was 20 cm long and 5 cm in diameter. Magnetic fields in the range -50 mG to +50 mG could be produced. For the reaction in an ethanol water mixture it is necessary to first active [IrCl(COD)(IMes)] with DMSO and H_2 prior to adding pyruvate.

S2. NMR characterization data

A range of experiments were carried out to characterize the products formed during and after the SABRE process. Samples were prepared with a fixed ratio of substrate to catalyst and the co-ligand DMSO which were dissolved in 0.6 mL of methanol-*d*$_4$ in a 5 mm NMR tube that was fitted with a J. Young's tap. The solutions were degassed by the freeze-pump-thaw technique and subsequently filled with *p*-H_2 gas to a 3 bar pressure. A typical ratio of catalyst, substrate, and co-ligand was 1:5:8 with the precatalyst loading being 5 mM. Fig. S1 portrays the hydride region of the resulting ^1H NMR spectrum at 245 K. It shows the presence of the three distinct iridium complexes. Fig. S2 reveals how these hydride ligand signals correlate with each other according to a 2D ^1H-^1H COSY NMR spectrum. As evident, the pair of hydride signals at δ −15.5 and δ −21.6 couple and are assigned to the product shown in Fig. S2 (blue triangles); signals at δ −14.9 and δ −24.0 results from the axial-equatorial bidentate spanning pyruvate (green circles); while signals as δ −27.2 and δ −29.1 are due to the equatorial-bidentate isomer (red stars). The latter two species therefore have the form [Ir(H)$_2$(η^2-pyruvate)(DMSO)(IMes)]. The structures for these products are indicated in Fig. S2.

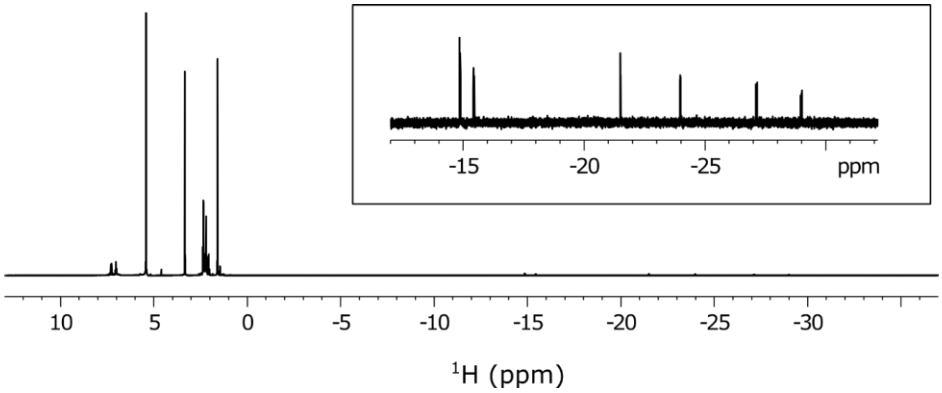

Fig. S1: ^{1}H NMR spectrum of a methanol-d_4 solution under 3 bar H_2 that contains the [IrCl(COD)(IMes)] pre-catalyst, DMSO and H_2 at 245 K. The inset trace shows the hydride region which confirms the complexity of this reaction.

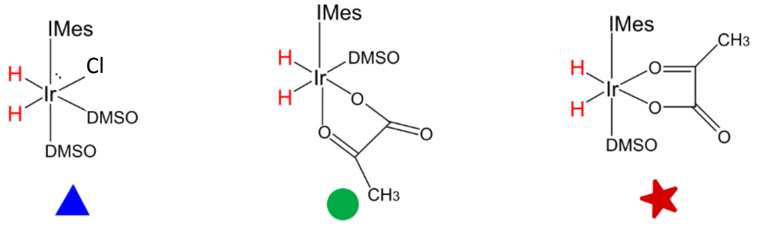

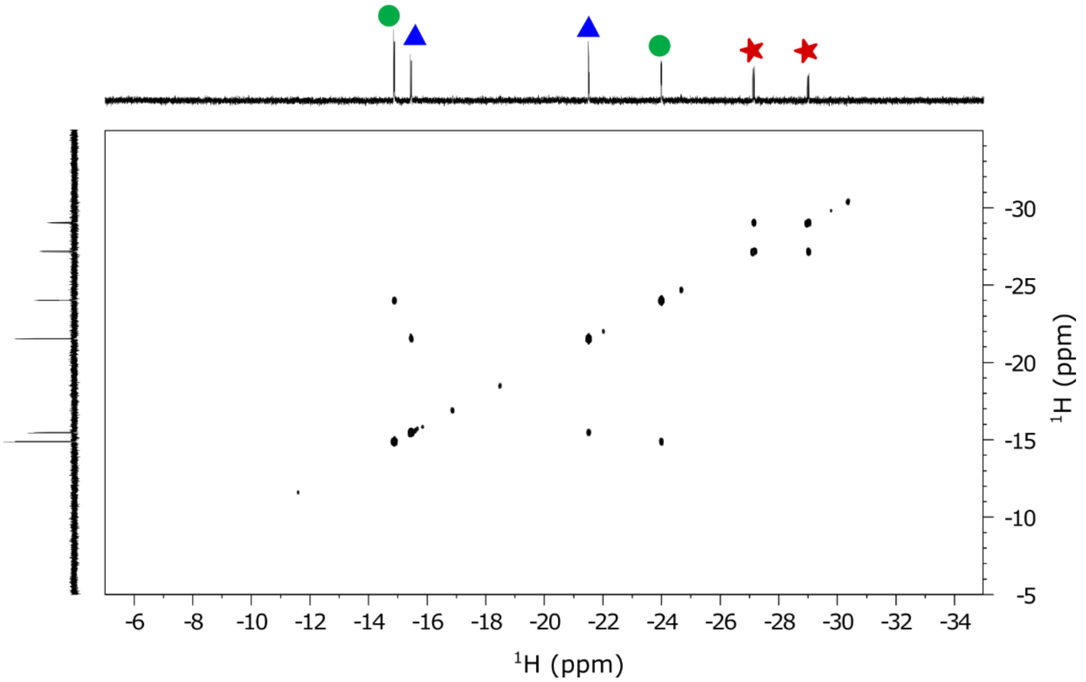

Fig. S2: ^{1}H-^{1}H COSY NMR spectrum of the solution used in Fig. S1 showing correlations between the key hydrides resonances whose structures are depicted above (colour coded).

Fig. S3 shows a one scan ^{1}H hyperpolarized NMR spectrum of this solution under SABRE. It was recorded at room temperature after adding p-H_2. The hydride ligands of $[Ir(H)_2(\eta^2\text{-pyruvate})(DMSO)(IMes)]$ at δ −27.2 and δ −29.1 exhibit PHIP.

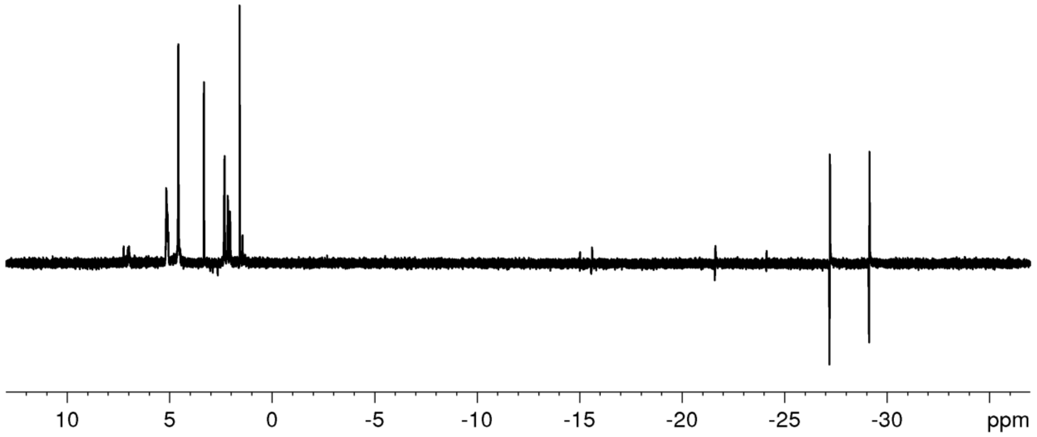

Fig. S3: ^{1}H NMR spectrum of the resulting methanol-$d_4$solution formed under 3 bar of p-H_2 and consisting of [IrCl(COD)(IMes)] precatalyst, **3** and DMSO at 298 K; hydride signals for the active species (equatorial-bidentate form (red stars)) dominate.

In the following section we characterise the complex, [Ir(H)$_2$(η^2-pyruvate)(DMSO)(IMes)], that is formed during the SABRE process (as denoted by red stars in Fig. S2) as illustrated in Scheme S1. These results are summarized in Table S1.

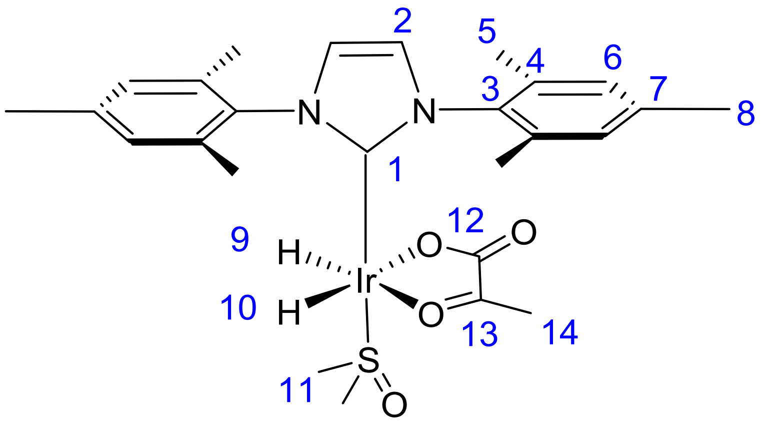

Scheme S1: Structure of [Ir(H)$_2$(η^2-pyruvate)(DMSO)(IMes)] where the labels refer to the resonance numbers of Table S1.

Table S1: NMR characterization data of [Ir(H)$_2$(η^2-pyruvate)(DMSO)(IMes)] of Scheme S1 (red stars) at 245 K.

**Resonance**	**^{1}H**	**^{13}C**
1	-	160.30
2	7.28	122.67
3	-	139.80
4	-	136.90
5	2.05, 2.16	16.61, 16.68
6	6.99, 7.05	128.21, 128.77
7	-	139.65
8	2.35	19.73
9	-27.15/-29.03 (d, $^2J_{HH}$ = 10 Hz)	-
10	-27.15/-29.03 (d, $^2J_{HH}$ = 10 Hz)	-
11	3.09	47.25
12	-	168.55
13	-	206.41
14	2.08	26.54

The second product formed contains no pyruvate and instead has DMSO and chloride bound to the metal centre (as denoted by blue triangles in Fig. S2). It is exemplified in Scheme S2 and the corresponding NMR data is summarized in Table S2.

Scheme S2: Structure of [IrCl(H)$_2$(DMSO)$_2$(IMes)] where the labels refer to the resonance numbers of Table S2.

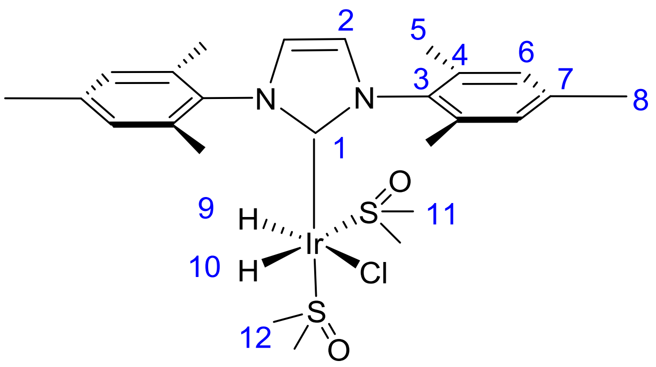

Table S2: NMR characterization data of the complex [IrCl(H)$_2$(DMSO)$_2$(IMes)] of Scheme S2 (blue triangles in Fig. S2) at 245 K.

Resonance	^{1}H	^{13}C
1	-	152.49
2	7.24	123.42
3	-	135.22
4	-	136.49/137.99
5	2.20	17.55/18.34
6	7.02	128.56
7	-	138.43
8	2.37	19.95
9	-21.53 (d, $^2J_{HH}$ = 6 Hz)	-
10	-15.47 (d, $^2J_{HH}$ = 6 Hz)	-
11	2.83, 3.12	48.77, 39.85
12	3.27, 3.19	43.74

The third complex involves axial-equatorial bidentate spanning pyruvate (green circles in Fig. S2) as shown in Scheme S3 and the corresponding NMR characterization data are summarized in Table S3.

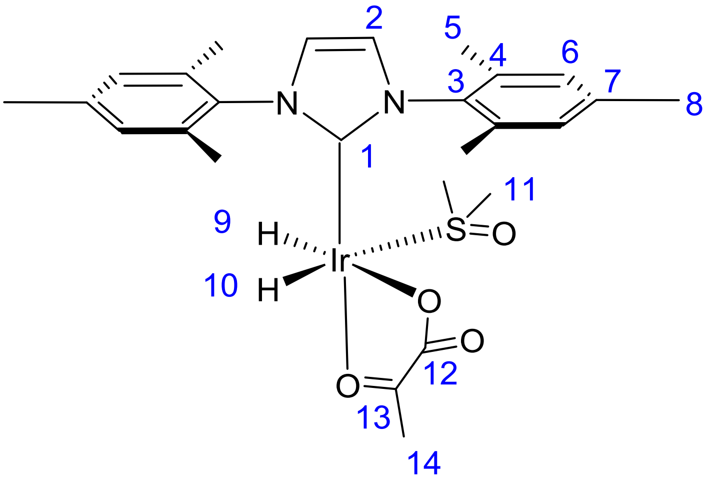

Scheme S3: Structure of [Ir(H)$_2$(η^2-pyruvate)(DMSO)(IMes)] where the labels refer to the resonance numbers of Table S3.

Table S3: NMR characterization data of the complex [Ir(H)$_2$(η^2-pyruvate)(DMSO)(IMes)] of Scheme S3 (green circles in Fig. S2) at 245 K.

Resonance	^{1}H	^{13}C
1	-	153.89
2	~7.2 (overlap)*	~120-130 (overlap)*
3	-	~120-130 (overlap)*
4	-	~120-130 (overlap)*
5	2.01, 2.24	16.63, 17.13
6	6.88, 7.13	128.19, 129.41
7	-	~120-130 (overlap)*
8	2.35	18.12
9	-24.01 (d, $^2J_{HH}$ = 7 Hz)	-
10	-14.89 (d, $^2J_{HH}$ = 7 Hz)	-
11	3.38	46.49
12	-	166.40
13	-	196.50
14	1.64	19.92

*This is the minor species present in solution and subsequently there is resonance overlap.

S3. Theory and simulations

SABRE is a magnetic resonance phenomenon that can be modelled quantum mechanically. There are two key aspects to the presented work, hyperpolarization build-up and the occurrence of singlet order within the substrate spins. Whilst a full theoretical study of SABRE involving all the spins of catalyst and substrate is beyond the scope of this work, approximations can be made to simplify the scenario. We consider a 4-spin coupled network as shown in Fig. S4.

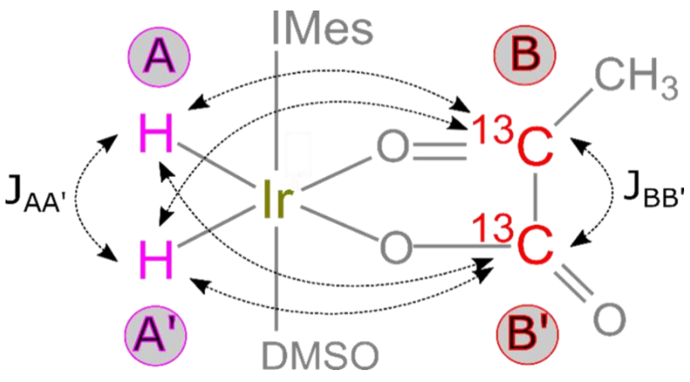

Fig. S4: Spin network showing an AA’BB’ model system.

The metal complex comprises two hydride protons and two ^{13}C nuclei from the pyruvate molecule. It can be considered as an AA’BB’ spin system as depicted in Fig. S4. Polarization transfer from p-H_2 to the ^{13}C nuclei occurs upon fulfilling the now detailed matching conditions. The density matrix

formalism was used to obtain these results.[2] The Hamiltonian of such an AA'BB' system can be written as,

$$H = \nu_A(I_{Az} + I_{A'z}) + \nu_B(I_{Bz} + I_{B'z}) + J_{AA'}\,\boldsymbol{I}_A.\boldsymbol{I}_{A'} + J_{BB'}\,\boldsymbol{I}_B.\boldsymbol{I}_{B'} + J_{AB}\,\boldsymbol{I}_A.\boldsymbol{I}_B + J_{AB'}\,\boldsymbol{I}_A.\boldsymbol{I}_{B'} + J_{A'B}\,\boldsymbol{I}_{A'}.\boldsymbol{I}_B + J_{A'B'}\,\boldsymbol{I}_{A'}.\boldsymbol{I}_{B'}$$

where ν_A and ν_B denote the chemical shifts of the hydride ligands (A, A') and the two carbon-13 spins (B, B') respectively. As written below, singlet-triplet basis sets conveniently characterises this Hamiltonian.[2b]

$$S_0 = \frac{1}{\sqrt{2}}(|\alpha\beta\rangle - |\beta\alpha\rangle);\ T_+ = |\alpha\alpha\rangle;\ T_0 = \frac{1}{\sqrt{2}}(|\alpha\beta\rangle + |\beta\alpha\rangle);\ T_- = |\beta\beta\rangle;$$

Based on these 16 different spin states result for the AA'BB' spin system. Out of these 16 states, 10 can be identified as symmetric spin states with respect to spin exchange operators, while 6 states can be identified as asymmetric spin states:

Symmetric states: $S_0^A S_0^B, T_0^A T_0^B, T_+^A T_-^B, T_-^A T_+^B, T_+^A T_+^B, T_-^A T_-^B, T_0^A T_-^B, T_+^A T_0^B, T_0^A T_-^B, T_-^A T_0^B$;

Anti-symmetric states: $S_0^A T_0^B, T_0^A S_0^B, S_0^A T_+^B, T_+^A S_0^B, S_0^A T_-^B, T_-^A S_0^B$;

These classifications allow one to represent the full Hamiltonian of the system in an orderly fashion. Fig. S5 graphically represents the Hamiltonian in this basis set. The grid numbers 1-10 signify the symmetric bases whereas 11-16 signify the antisymmetric basis states. The 16 X 16 matrix portrays some very exciting features that we describe below. Each coloured block signifies a non-zero mathematical element whilst empty spaces represent a null.

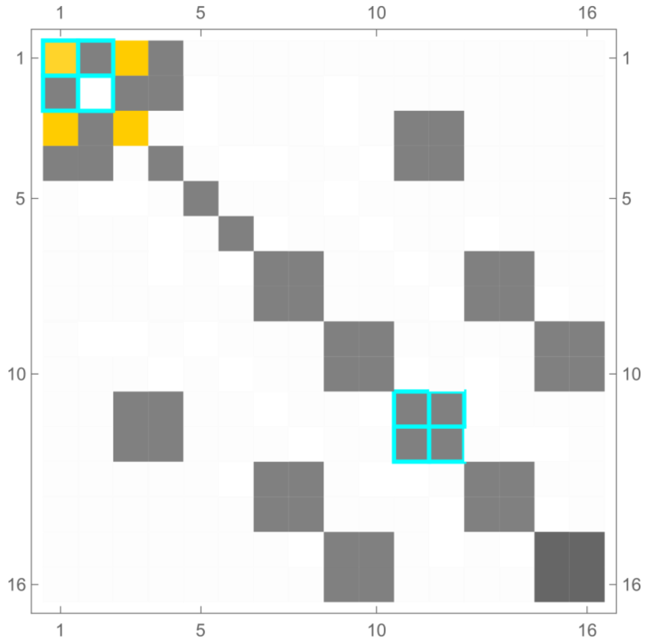

Fig. S5: Graphical illustration of the 16×16 matrix Hamiltonian in the singlet-triplet basis where numbers 1-10 on the axis symbolise symmteric basis states and 11-16 imply anti-symmteric basis states. These are listed in the same order as described in the text.

Since the initial state of the hydride ligands is created as pure singlet order, it is feasible to drive hyperpolarization transfer from them (A, A') to the target spins (B, B'). The exact transfer conditions can be calculated by examining two relevant subsets of the Hamiltonian matrix.

From the matrix block we can see that $S_0^A S_0^B$ can connect to $T_+^A T_-^B$ (and $T_-^A T_+^B$). This is portrayed by the yellow blocks of Fig. S5.

$$\begin{array}{c} \\ |S_0^A S_0^B\rangle \\ |T_+^A T_-^B\rangle \end{array} \begin{array}{c} \begin{array}{cc} |S_0^A S_0^B\rangle & |T_+^A T_-^B\rangle \end{array} \\ \begin{pmatrix} J_{AA'} + J_{BB'} & \frac{(J_{AB'} - J_{A'B'}) - (J_{AB} - J_{A'B})}{4} \\ \frac{(J_{AB'} - J_{A'B'}) - (J_{AB} - J_{A'B})}{4} & \nu_A - \nu_B - \frac{(J_{AB'} + J_{A'B'}) + (J_{AB} + J_{A'B})}{4} \end{pmatrix} \end{array}$$

In this case, transfer of population between the states takes place as the diagonal elements match each other thereby allowing off-diagonal terms to drive population flow from the hydrides to the target nuclei. This resonance condition can be written as,

$$\nu_A - \nu_B = \pm[J_{AA'} + J_{BB'} + \tfrac{(J_{AB'}+J_{A'B'})+(J_{AB}+J_{A'B})}{4}]$$

The positive sign is for $S_0^A S_0^B \leftrightarrow T_+^A T_-^B$, whilst the negative sign is for $S_0^A S_0^B \leftrightarrow T_-^A T_+^B$.

In the case of mono-labelled pyruvates (**1** and **2**), the resonance condition simply becomes,

$$\nu_A - \nu_B = \pm[J_{AA'} + \tfrac{(J_{AB}+J_{A'B})}{2}]$$

and the magnetic field to fulfil the resonance condition can further be written as,

$$B_0 = \pm[\frac{J_{AA'} + \frac{(J_{AB}+J_{A'B})}{2}}{\gamma_A - \gamma_B}]$$

The label A refers to hydride while B reflects the ^{13}C spin. Applying the values of our present system we calculate that an optimum field of between 9 and 3 mG is required to achieve the maximum level of polarization transfer in the target ^{13}C spins depending on whether **1** or **2** is involved.

Remarkably, the singlet state of pyruvate **3** gains significant population if the following conditions are filled. The following matrix subset illustrates this point (highlighted in coloured border in Fig. S5).

$$\begin{array}{c} \\ |S_0^A S_0^B\rangle \\ |T_0^A T_0^B\rangle \end{array} \begin{array}{c} \begin{array}{cc} |S_0^A S_0^B\rangle & |T_0^A T_0^B\rangle \end{array} \\ \begin{pmatrix} J_{AA'} + J_{BB'} & \frac{(J_{AB} - J_{A'B}) - (J_{AB'} - J_{A'B'})}{4} \\ \frac{(J_{AB} - J_{A'B}) - (J_{AB'} - J_{A'B'})}{4} & 0 \end{pmatrix} \end{array}$$

$|S_0^A T_0^B\rangle$ $|T_0^A S_0^B\rangle$

$$\begin{array}{c} |S_0^A T_0^B\rangle \\ |T_0^A S_0^B\rangle \end{array} \begin{pmatrix} J_{AA'} & \frac{(J_{AB} - J_{A'B}) - (J_{AB'} - J_{A'B'})}{4} \\ \frac{(J_{AB} - J_{A'B}) - (J_{AB'} - J_{A'B'})}{4} & J_{BB'} \end{pmatrix}$$

When the off-diagonal elements become comparable, it is possible to transfer magnetization between the singlet states of the hydride ligands (S_0^A) and the pyruvate (S_0^B) manifold. The matrix sub-block fulfils this criterion when $J_{AA'} \simeq \pm J_{BB'}$. Earlier reports on a $^{15}N_2$-diazirine based substrate satisfied this criterion naturally with $J_{15N\text{-}15N}$ = 17.8 Hz whilst J_{HH} = 8.0 Hz[2b] even though the match was not exact; singlets were formed efficiently with >4 orders of polarization enhancement. In our case a similar analogy can be drawn with pyruvate. Now the *J*-coupling within the carbon-13 pair is 62.5 Hz and significantly higher than the hydride coupling (8.0 Hz). Despite this apparent disparity in *J*-value, it is still possible to pump the singlet polarization into pyruvate, albeit with less efficiency.

S4. Examples of Hyperpolarized NMR spectra

Fig. S6 shows a series of NMR spectra that demonstrate hyperpolarized singlet order can be created in samples of 50:50 mixtures of **1** and **3** under three different scenarios. When a magnetic field of ~500 mG is experienced by the catalyst during transfer only singlet order is formed in **3** as detailed in Fig. S6a because of the mismatch for direct Zeeman transfer. Transfer at a very low, but non-zero field, again only sees singlet order formed because of a similar mismatch for Zeeman transfer. These deductions are confirmed by the characteristic 'down-down-up-up' pattern seen in the resulting NMR spectra. However, when the process occurs at a 9 mG field, both the singlet form and 'direct' magnetization transfer is detected (Fig. S6c), the consequence is both Zeeman and singlet transfer now operates efficiently. This particular experiment used a mixture of **1** and **3** to highlight this difference in polarization build-up pathway in a clear manor as predicted by the theory.

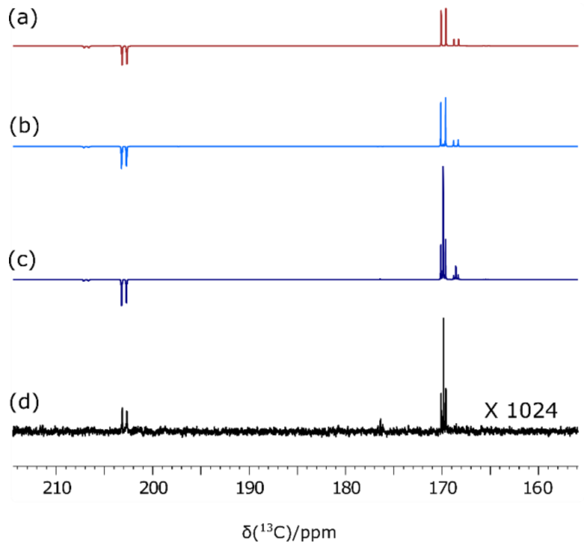

Fig. S6: SABRE hyperpolarized ^{13}C NMR spectra of 50:50 mixture of **1** and **3** recorded after transfer (a) at the Earth's magnetic field, (b) inside a mu- metal shield (~0 G) and (c) optimized SABRE-SHEATH condition (~9 mG) with the corresponding (d) thermal signal (vertically scaled by 1024 times) at 11.75 T spectrometer for comparison.

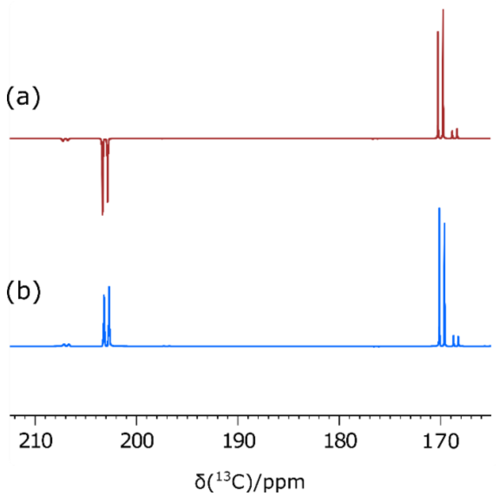

Fig. S7: SABRE hyperpolarized ^{13}C NMR spectra of **3** detected after (a) a 90˚ pulse and (b) refocusing by a suitable pulse-sequence.

Fig. S7 shows a fully in-phased singlet derived spectrum that was achieved by suitable refocussing. The efficiency of singlet order build-up depends on several factors but most importantly on the duration of *p*-H_2 bubbling. Fig. S8 shows a stacked plot NMR spectrum alongside their integrated intensity values as a function of the *p*-H_2 bubbling duration. Optimum enhancement levels are achieved after 50 sec. of continuous bubbling. This significantly long build-up time can be attributed to the resonance mismatch condition as described in the theory section and the slow exchange dynamics of the system.

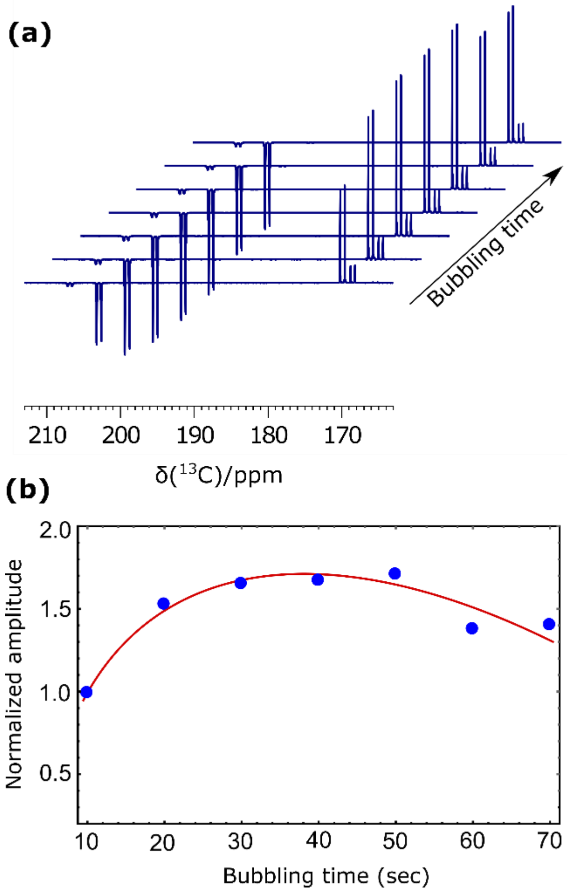

Fig. S8: (a) Series of hyperpolarized ^{13}C NMR spectra of **3** as a function of p-H_2 bubbling time and (b) respective normalised integral values from these spectra showing that a maximum polarization level is achieved after ~ 50 sec. of p-H_2 bubbling.

Fig. S9 shows the corresponding field plot for agent **2** as a function of the magnetic field experienced by the sample during transfer. A maximum enhancement level is achieved at field of −3.3 mG when compared to that for **1** which is detailed in Fig. 2 of the main paper.

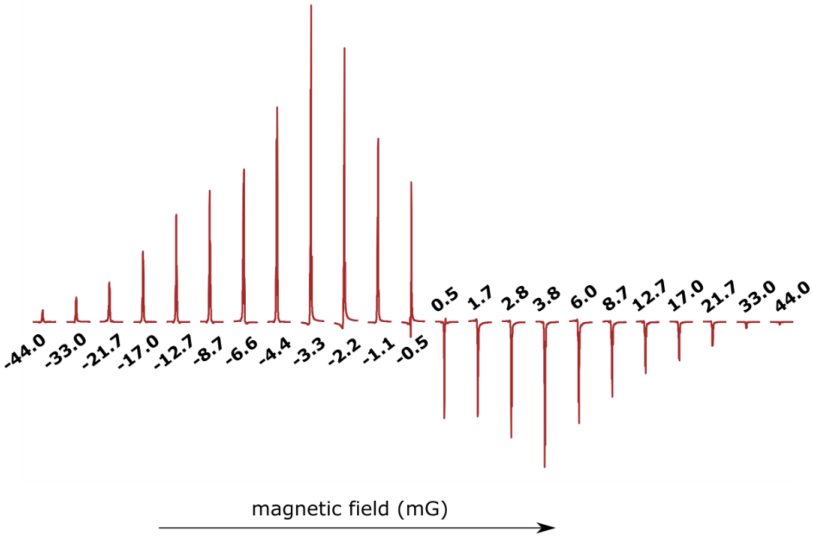

Fig. S9: Peaks extracted from a series of hyperpolarized ^{13}C SABRE NMR spectra of **2** as a function of the magnetic field experienced during transfer, from −44 mG to +44 mG. The maximum signal strength is achieved at −3.3 mG.

Fig. S10 shows the resulting single scan response of an unlabelled sample of **4** at a 0.2 mM loading (2.5 mg in 0.6 ml of CD_3OD)**.** The resulting signal to noise ratio was 280 for the 1-^{13}C resonance after transfer at 6 mG, with signals for 2-^{13}C and 3-^{13}C also being clearly visible. We note for clarity that the corresponding 3-^{13}C signal was also visible in the NMR spectra of samples **1-3** referred to earlier. These results further highlight the context of the successful hyperpolarization of pyruvate as measurement under normal, Boltzmann controlled, conditions would require ~30 hrs to match these results.

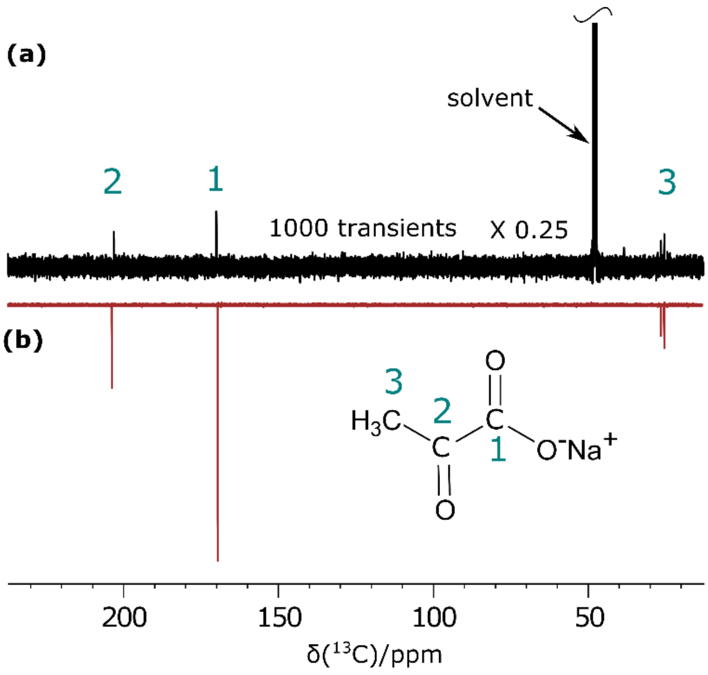

Fig. S10: $^{13}C\{^1H\}$ NMR spectra of **4** measured at 11.75 T: (a) thermally polarized spectrum after 1000 signal additions recorded over 16 hours. (b) single scan hyperpolarized SABRE-SHEATH result after polarization transfer at 6 mG. The ready detection of all three pyruvate carbon signals is revealed.

S5. Mu-metal shielded solenoid

In order to collect the low field data, a low magnetic field system was designed and developed as part of this work. The system comprises a solenoid, a mu-metal shield, current limiting resistors, a DC power supply and a DC milli-Gauss (mG) meter. The solenoid coil was built around a 3D printed solenoid base of 210 mm in length, 14.25 mm outer diameter and 10 mm inner diameter. The coil consists of 390 turns (N) (wire gage 0.5 mm with 25 µm varnish coating), with 7.5 mm radius and 200 mm in length (l). DC measurement on the solenoid showed a 130 µH inductance and a 1.55 Ω resistance. In this system the solenoid is placed inside a zero-magnetic field mu-metal shield. The stray magnetic field inside the mu-metal shield was measured to be <0.5 mG. The mu-metal shield used in our experiments features a wall thickness of 1 mm with external diameter of 30 mm and length of 28 mm.

When including the shunt, the total resistance is about 940 Ω ± 2 %. This added resistance is set to limit the current passing through the solenoid and allow the generation of very low magnetic fields (in the mG range). These can be adjusted using relatively high voltage levels (up to 5 V). The DC resistance of the solenoid coil along with this added resistor acts like a voltage divider leading to a relatively low voltage drop across the coil with respect to the voltage at the power supply output due to the small resistance of the coil (1.55 Ω) as illustrated in Fig. S11.

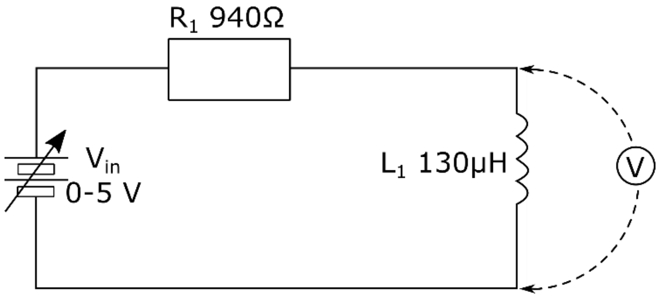

Fig. S11: The electrical circuit used to generate the desired magnetic field.

The voltage across the coil is given as,

$$V = V_{in}(\frac{R_{L1}}{R_{L1} + R_1})$$

Where V_{in} is the power supply output, R_{L1} is the coil DC resistance and R_1 is the added resistance. The current passing through the coil is can be easily calculated by,

$$I = \frac{V}{R_{L1}}$$

In this case for the maximum 5 V supply voltage the voltage across the coil is equal to 8.23 mV and the maximum current passing through the coil is 5.3 mA (*i.e.* max coil power is 44 μW).

The power supply used for this application is KEITHLEY 2231A-30-3 triple independent channel DC power supply. Two channels can supply up to 30 V at 3 A each and a third channel can provide up to 5V at 3 A. This system is a linear based design with less than 5mV p-p noise per output. The system features setting and reading resolution of 10 mV and 1 mA with voltage accuracy of ≤ 0.06 %+20 mV and current accuracy of ≤ 0.2 %+10 mA. For our application we have used the 0 to 5 V channel with both forward and reverse polarity to achieve positive and negative magnetic fields respectively. The power supply and mG meter used in this study are shown below.

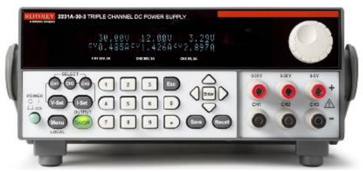

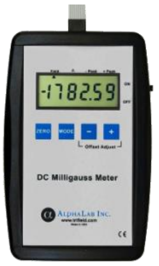

The shielded solenoid is connected to one of the power supply channels through the added resistor while the DC mG meter probe is inserted in the centre of the solenoid to measure the magnetic field in the Z direction. By adjusting the voltage applied and hence the current through the solenoid the magnetic field inside the shielded solenoid can be adjusted to any value between −110 to +110 mG with resolution (SR) of 1 mG. It is worth noting here that the field resolution is controlled by the size of the resistor R. The higher the added resistance, the smaller the field resolution (*i.e.* SR α 1/R). The added resistor also affects the maximum magnetic field. For a given maximum magnetic field B_{max} increasing the value of the added resistor requires a higher voltage (*i.e.* V_{max} α R for a given B_{max}).

The magnetic field B inside the shielded solenoid was measured relative to the applied voltage and current (I). Results showed perfect overlap with the theoretically calculated values which were calculated using the equation as illustrated in Fig. S12 where μ is the permeability.

$$B = \frac{\mu l N}{I}$$

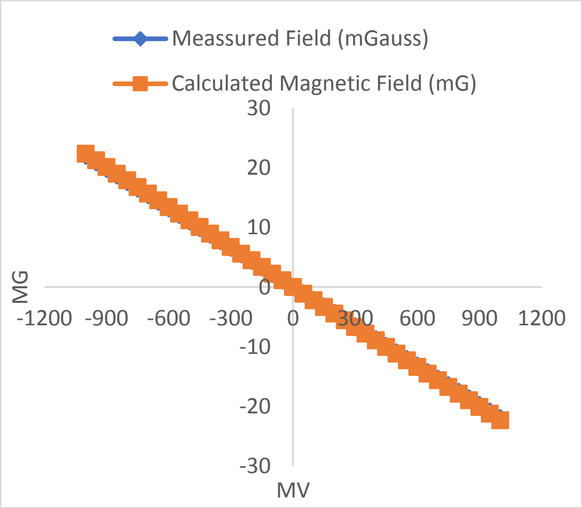

Fig. S12: Plot showing measured magnetic field in mG as a function of externally applied voltage (mV) to the solenoid. Theoretically calculated values (orange) show excellent match with experimentally measured numbers (blue).

Fig. S13 shows the whole set-up to carry out SABRE and SABRE-SHEATH experiments in a systematic fashion.

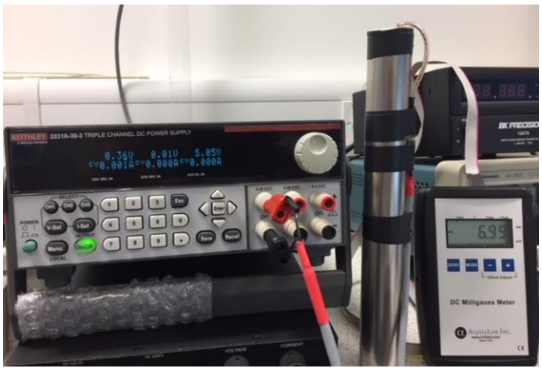

Fig. S13: A voltage controlled low-magnetic field set-up developed to carry out SABRE hyperpolarization experiments.

S6. References

[1] L. D. Vazquez-Serrano, B. T. Owens, J. M. Buriak, *Inorg. Chim. Acta* **2006**, *359*, 2786-2797.

[2] a) J. Natterer, J. Bargon, *Prog. Nucl. Magn. Reson. Spectrosc.* **1997**, *31*, 293-315; b) T. Theis, G. X. Ortiz, A. W. Logan, K. E. Claytor, Y. Feng, W. P. Huhn, V. Blum, S. J. Malcolmson, E. Y. Chekmenev, Q. Wang, W. Warren, *Sci. Adv.* **2016**, *2*, e1501438.
